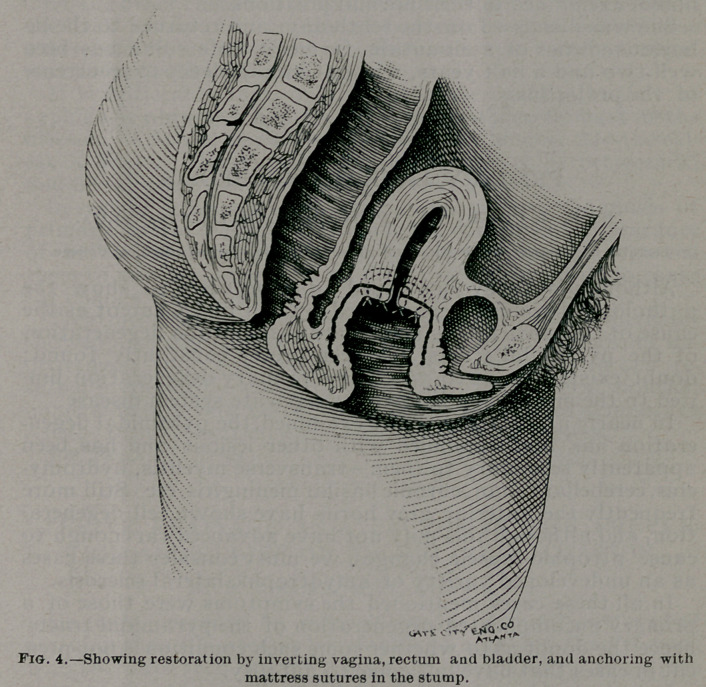# Hypertrophic Elongation of the Cervix Uteri, with Complete Eversion of Vagina, from Fibroid Tumor of the Cervix, Restoration by Operation*Read before the Southern Surgical and Gynecological Association, at [Nashville, Tenn., Nov. 10-12, 1896.

**Published:** 1897-02

**Authors:** George Henry Noble

**Affiliations:** Atlanta, Ga., President of the Medical Association of Georgia; Secretary of the Section of Obstetrics and Gynecology, American Medical Association; and Gynecologist to the Grady Hospital, Atlanta, Ga.; 186 South Pryor Street


Southern Medical Record.

A MONTHLY JOURNAL OF MEDICINE AND SURGERY.

VOL. XXVII. ATLANTA, GA., FEBRUARY, 1897. No. 2

### Original Articles.

### HYPERTROPHIC ELONGATION OF THE CERVIX UTERI, WITH COMPLETE EVERSION OF VAGINA, FROM FIBROID TUMOR OF THE CERVIX. RESTORATION BY OPERATION.*

BY GEORGE HENRY NOBLE, M. D., ATLANTA, GA.,

President of the Medical Association of Georgia; Secretary of the Section of Obstetrics and Gynecology, American Medical Association; and Gynecologist to the Grady Hospital, Atlanta, Ga.

Mrs. H., a tall countrywoman, came to me from the mountainous region of Georgia on January 25th, 1894. She stated that shortly after her first confinement, which occurred eight and a half years ago, she discovered a tumor, about the size of an ordinary marble, growing upon the neck of her womb, which, after a subsequent childbirth, two and a half years later, had grown to the size of a hen's egg. From this time she dates the prolapsus, the tumor having escaped from the vagina. She had not suffered much, except from the discomfort of aching, dragging, etc. Six years ago the lower extremity of the tumor ulcerated, but afterward healed, and two months before admission ulcerated again, from which she suffered the effects of absorption—accelerated pulse, elevated temperature, chills, profuse diaphoresis, etc.—until she became anemic and more or less emaciated. The family history was good.

The condition on admission was as follows: Temperature, 102⅗°; pulse, 116, weak and compressible; respiration, 22 per

*Read before the Southern Surgical and Gynecological Association, at Nashville, Tenn., Nov. 10-12, 1896.

FIG 1.—Showing tumor and everted vagina before operation.

minute; menstruation normal; bowels recently constipated; painful and frequent urination, and at times incontinence of urine. The urinary analysis disclosed nothing abnormal, except a diminished excretion of urea. Vaginal discharges profuse and muco-purulent in character. Heart, lungs, etc.,

FIG. 2.—Sectional view of same.

negative. Inspection shows an egg-shaped fibroid tumor about nine inches long and six inches in diameter, attached by a pedicle on its side to the upper right margin of the os uteri. There are five superficial ulcers, three upon the anterior and two upon the posterior surface of the tumor, averaging the size of a silver dollar. The vagina is completely everted, not even a sulcus remaining within the vulva. The bladder and rectum are turned out through the vaginal orifice, forming deep pouches in front of and behind the mass. The cervix is as large as an ordinary-sized man's wrist, and elongated sufficiently to permit extrusion of the vagina, while the fundus uteri is near its normal position. The cavity of the uterus is three inches deep, that of the cervix seven inches, making a total of ten inches. The round ligaments are very much hypertrophied, feeling like cords larger than a lead pencil. Other pelvic organs seem free from disease.

FIG. 3.—Dissection of bladder and rectum from cervix uteri, and amputation of latter at the internal os.

As hysterectomy is often unsatisfactory in procidentia uteri, I determined to do a supravaginal amputation of the neck and replace the everted vagina, knowing that the supports were equal to the task of sustaining the parts.

The operation consisted in the usual circular incision dissecting off the bladder, rectum, and post-uterine peritoneum from the cervix for a distance of six and a half inches, and amputation of the cervix at the internal os, ligating the uterine arteries and other large vessels as they were encountered. The parts were very vascular; many of the vaginal branches were as large as small quills; consequently, the operation was tedious, frequent ligation being necessary. After removal of the neck of the womb, the vagina was stitched to the stump by four mattress sutures, introduced an inch back of the free margin of the vagina, then through the walls of the uterus, beneath the cut surface of the stump, emerging from the cervical canal, then passed in the reverse direction and tied on the

**FIG. 4.—Showing restoration by inverting vagina, rectum and bladder, and anchoring with mattress sutures in the stump.**

surface of the vagina. These sutures served the double purpose of closely applying the vagina to the stump, especially in the angles of the womb, and of controlling oozing from the uterus. The margins of the vagina were fastened by superficial sutures to the mucous membrane of the cervical canal, and the remainder of the womb closed in a transverse line, except at the angles where drainage was provided for. The vagina was dressed with gauze to prevent pouching or pocketing between the opposing raw surfaces. Thirty-eight minutes were consumed in performing the operation.

She made a very fair recovery, considering her condition at the time of the operation. The suppurating tumor having been removed, the temperature dropped, but on the seventh day, when the mattress sutures cut into the tissues, the temperature reached 103°, but came down immediately on removal of the stitches. From that time on her recovery was uninterrupted. The uterus mounted higher in the pelvis, the vaginal orifice contracted to some extent, and the vagina, rectum and bladder remained in their normal positions.

She was dismissed on the tenth day, and returned to the laborious duties of a mountain farmer's wife. She has been well two and a half years, without any tendency to recurrence of the prolapsus.

*186 South Pryor Street.*

## Figures and Tables

**Fig 1. f1:**
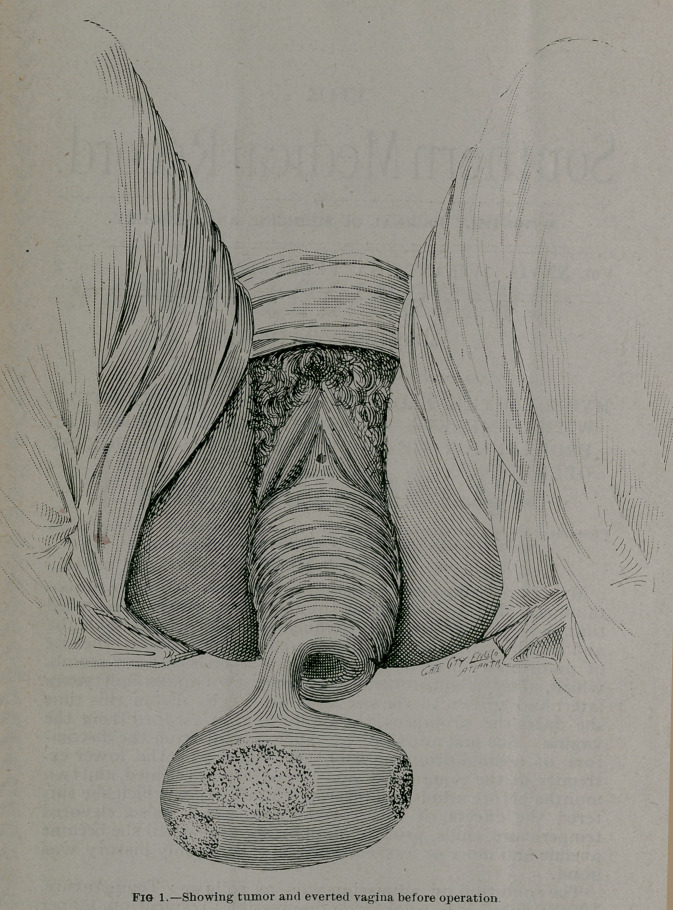


**Fig. 2. f2:**
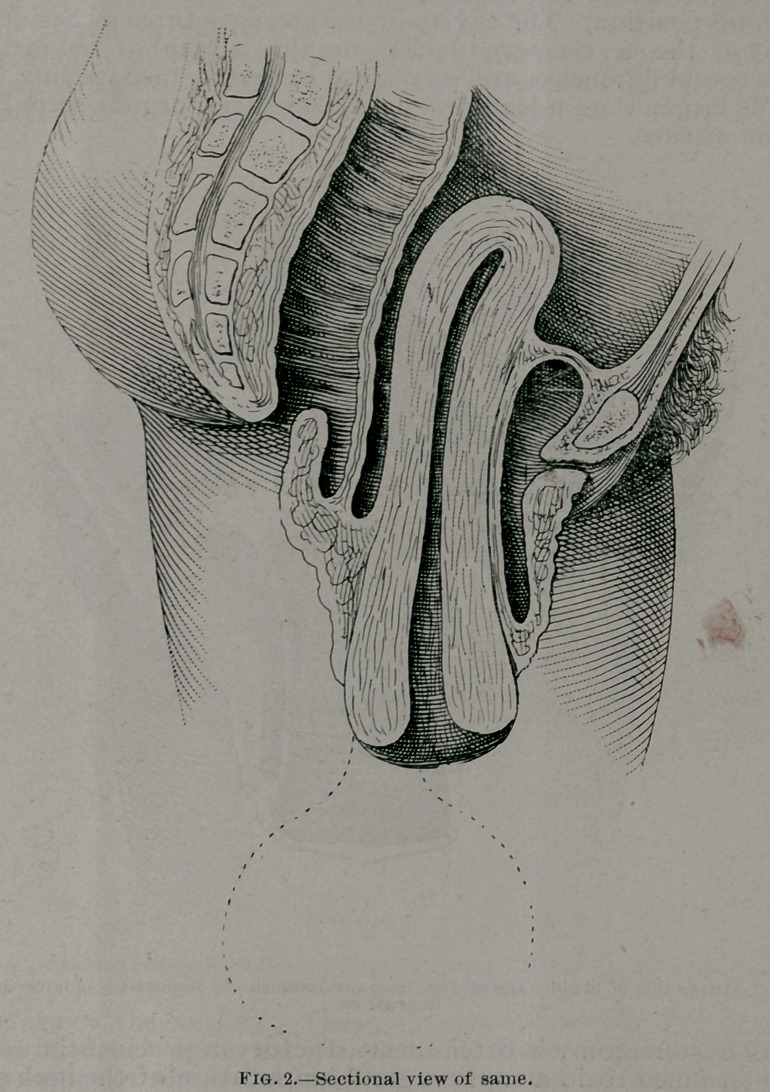


**Fig. 3. f3:**
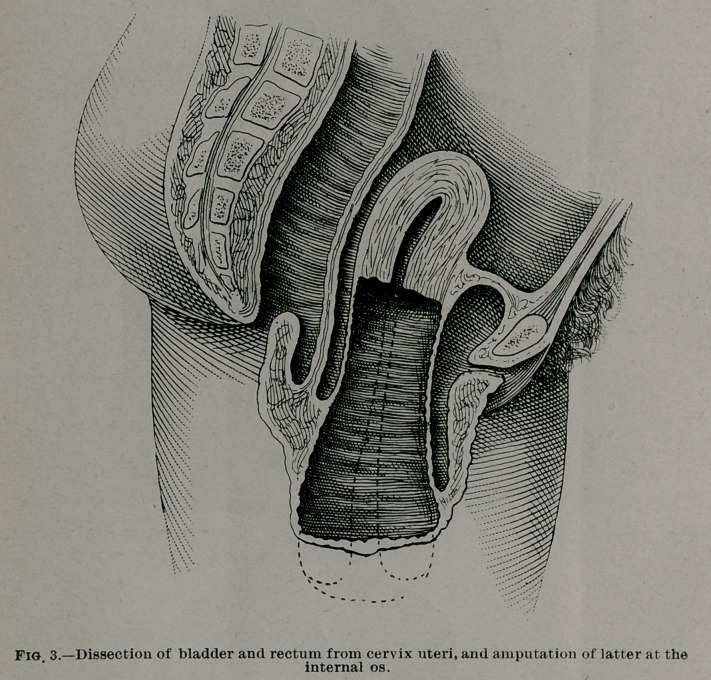


**Fig. 4. f4:**